# Insulin receptor substrate family member IST-1 regulates the development of *Caenorhabditis elegans* *age-1* and *aap-1* mutants

**DOI:** 10.17912/micropub.biology.001580

**Published:** 2025-06-25

**Authors:** David Guerrero-Gómez, Juan Cabello, Antonio Miranda-Vizuete

**Affiliations:** 1 Redox Homeostasis Group, Instituto de Biomedicina de Sevilla, Hospital Universitario Virgen del Rocío/CSIC/Universidad de Sevilla, Sevilla, Spain; 2 Center for Biomedical Research of La Rioja, Logroño, La Rioja, Spain

## Abstract

Insulin receptor substrate (IRS) is a class of adaptor proteins that mediate the activation of transmembrane tyrosine kinase receptors to downstream effectors. The IST-1 protein is the sole IRS present in *Caenorhabditis elegans ,* which has been poorly studied in this animal model. Here, we show that *ist-1* mutants develop normally but exhibit sterility, larval arrest and dauer phenotypes when combined with mutations in *age-1* and *aap-1* genes, which encode the catalytic and regulatory subunits of phosphatidylinositol 3-kinase (PI3K), respectively. In contrast, no major genetic interactions are observed with mutations in other genes of the worm insulin pathway, either upstream or downstream AGE-1 / AAP-1 . We conclude that IST-1 , the only IRS in *C. elegans* , functions as a positive regulator of PI3K in the canonical insulin pathway during development.

**Figure 1. Developmental and genetic interactions of ist-1 and insulin pathway mutants in Caenorhabditis elegans:**
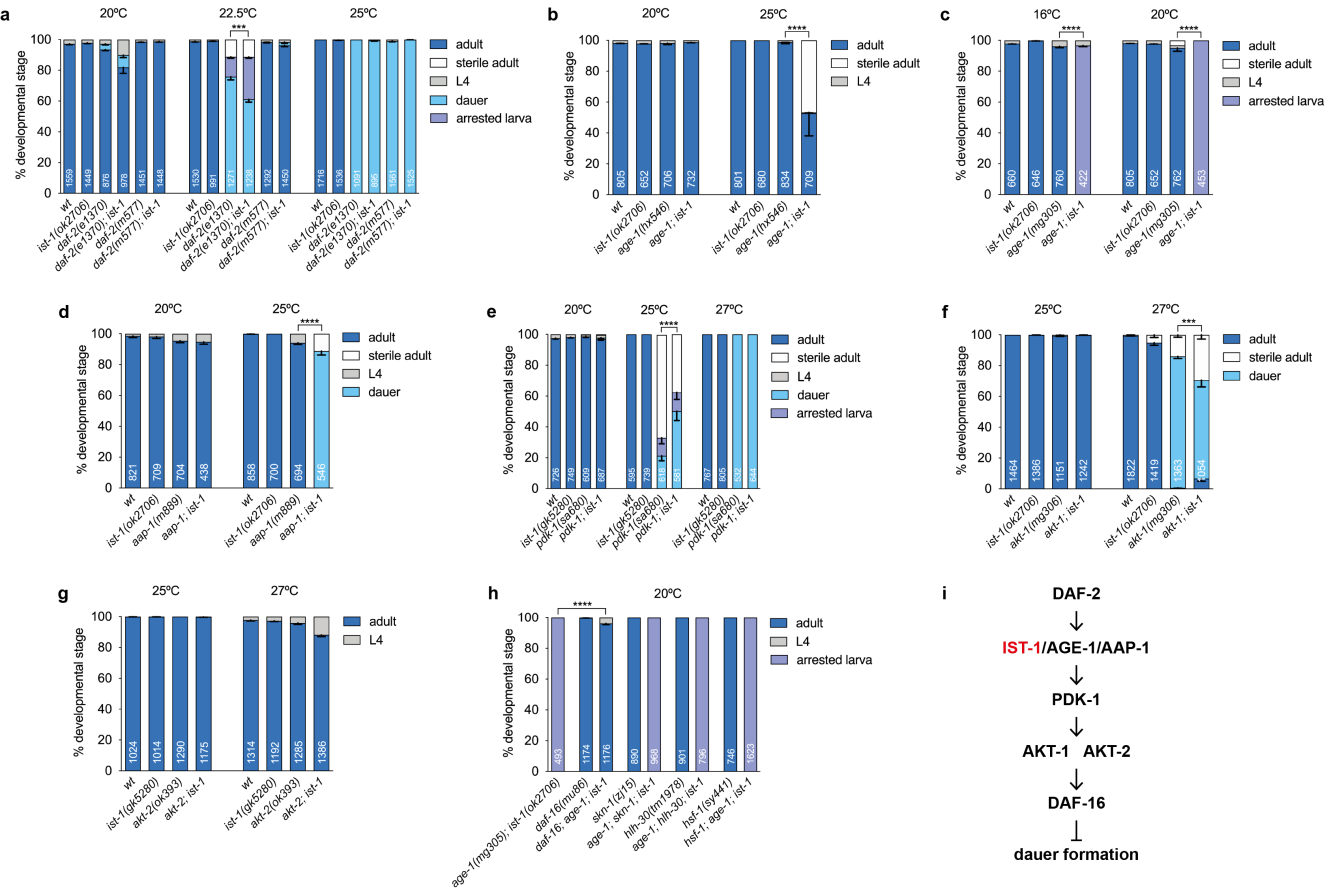
Developmental stage distribution of double mutant combinations of *ist-1 ( ok2706 )* , *ist-1 ( gk5280 )* or *age-1 ( mg305 ); ist-1 ( ok2706 )* worms with the following alleles **a)** *daf-2 ( e1370 )* and *daf-2 ( m577 )* ; **b)** *age-1 ( hx546 )* ; **c)** *age-1 ( mg305 )* ; **d)** *aap-1 ( m889 )* ; **e)** *pdk-1 ( sa680 )* ; **f)** *akt-1 ( mg306 )* ; **g)** *akt-2 ( ok393 )* and **h)** *daf-16 ( mu86 )* , *skn-1 ( zj15 )* , *hlh-30 ( tm1978 )* and *hsf-1 ( sy441 )* . Data are the mean +/- SEM from three independent experiments, each with three biological replicates. Numbers indicate the total of scored animals. *** p<0.001; **** p<0.0001 by 2way ANOVA with Tukey´s multiple comparison test. Animals were grown at the indicated temperatures and incubated for 3 to 6 days, depending of the temperature, to allow full development and accurate assessment of the different stages. The *ist-1 ( gk5280 )* allele was used in double mutant combinations with *pdk-1 ( sa680 )* and *akt-2 ( ok393 )* , both located on LGX, to facilitate the isolation of recombinants. **i)** Schematic representation of the insulin pathway with IST-1 acting downstream DAF-2 at the level of AGE-1 / AAP-1 .

## Description

The evolutionarily conserved insulin and insulin-like growth factor 1 (IGF1) signaling pathway (IIS) regulates numerous aspects of organismal metabolism, development, cell and organ growth, stress resistance, lifespan or memory among other traits (Murphy and Hu 2013, White and Kahn 2021). Binding of insulin/IGF1 peptides to their receptors activates a cascade of kinase proteins that ultimately converge on a group of transcription factors, mainly belonging the FoxO family, which coordinate the transcriptional output of the pathway (Huang and Tindall 2007). Signal transduction from the activated insulin receptors to the downstream effectors is mediated by a class of adaptor proteins known as insulin receptor substrate (IRS), of which six members are present in mammals (IRS1-6) whereas only one is found in *Drosophila melanogaster* (CHICO) or *Caenorhabditis elegans* ( IST-1 ) (Bohni, Riesgo-Escovar et al. 1999, Wolkow, Munoz et al. 2002, Shaw 2011). IRS proteins lack intrinsic enzymatic activity and instead function as scaffold that facilitate the assembly of signaling complexes (Shaw 2011). Despite the extensive molecular and functional characterization of the *C. elegans* insulin pathway, it is surprising that only two studies have investigated the function of the sole IRS orthologue IST-1 in the nematode (Wolkow, Munoz et al. 2002, Cheng, Lee et al. 2022).

While best known for its role in regulating dauer larva development and lifespan, the *C. elegans* DAF-2 /insulin receptor pathway also has additional developmental functions, revealed by distinct *daf-2* mutations and double mutant combinations. Phenotypes associated with *daf-2* mutations include embryonic arrest, arrest at different larval stages, and the production of sterile adults (Gems, Sutton et al. 1998). Downstream *daf-2* , the genes *age-1* and *aap-1* encode the catalytic and regulatory subunits, respectively, of phosphatidylinositol 3-kinase (Morris, Tissenbaum et al. 1996, Wolkow, Munoz et al. 2002), which catalyzes the conversion of phosphatidylinositol 4,5-biphosphate (PIP2) to phosphatidylinositol 3,4,5-triphosphate (PIP3) that serves as a key signaling molecule to activate the downstream kinase PDK-1 (Paradis, Ailion et al. 1999). The *age-1 ( hx546 )* allele is the weakest in the *age-1* allelic series and only produces dauers at 27ºC (Malone, Inoue et al. 1996), whereas *age-1 ( mg44 )* , the strongest allele of the series, causes a constitutive dauer phenotype at all temperatures (Gottlieb and Ruvkun 1994) that is suppressed by the *akt-1 ( mg144 )* gain-of-function mutation in the downstream AKT-1 kinase (Paradis and Ruvkun 1998).

Wolkow et al. reported that *ist-1* RNAi downregulation in *age-1 ( hx546 )* mutants at 25.5ºC increases the number of dauer larvae, suggesting that IST-1 functions within the insulin pathway (Wolkow, Munoz et al. 2002). Moreover, *ist-1* RNAi downregulation in an *age-1 ( mg44 ); akt-1 ( mg144 )* double mutant partly restored the dauer phenotype at 25.5ºC, leading the authors to propose that IST-1 may act in a parallel branch downstream of the DAF-2 insulin receptor (Wolkow, Munoz et al. 2002). This hypothesis was previously anticipated by Paradis and Ruvkun, who observed that *akt-1 ( mg144 )* mutants failed to suppress the dauer-constitutive phenotype of *daf-2 ( e1370 )* mutants at 25ºC (Paradis and Ruvkun 1998). Further work subsequently identified this parallel branch downstream DAF-2 as the RAS signaling pathway (Nanji, Hopper et al. 2005).

Additional indirect evidence supporting a role for IST-1 in dauer formation via the insulin pathway was provided by the finding that *ist-1* expression is induced in *dpy-11* mutants, which were isolated in a genetic screen for dauer regulatory genes that modulate the activity of the FoxO transcription factor DAF-16 (Dumas, Delaney et al. 2013). More recently, IST-1 has been shown to function in *C. elegans* AWC neurons mediating aversive olfactory learning through the DAF-2 c isoform (Cheng, Lee et al. 2022). Notably, a transgenic strain expressing eGFP under the control of a 14.8 kb *ist-1* promoter fragment revealed strong expression in several head neuron pairs , including AWC, ASE, ASG, ASH, ASI, ASK, BAG, RIC, AUA, AIM and RIG (Cheng, Lee et al. 2022), many of which are known to regulate dauer formation (Bargmann and Horvitz 1991).

Given the synthetic dauer phenotypes reported with *ist-1* RNAi downregulation in *age-1 ( hx546 )* mutants (Wolkow, Munoz et al. 2002) and the IST-1 expression in neurons involved in regulating dauer development (Cheng, Lee et al. 2022), we investigated the role of IST-1 in dauer formation through the insulin signaling pathway using the *ist-1 ( ok2706 )* loss-of-function allele (Cheng, Lee et al. 2022). Single *ist-1 ( ok2706 )* mutants did not produce dauers at 20ºC, 22.5ºC or 25ºC (Figure 1a) and double mutants combining *ist-1 ( ok2706 )* with class 1 *daf-2 ( m577 )* and class 2 *daf-2 ( e1370 )* weak alleles (Patel, Garza-Garcia et al. 2008) did not show enhanced dauer formation at 20ºC, nor did suppress *daf-2* constitutive dauers at 25ºC (Figure 1a). Only the dauers generated by *daf-2 ( e1370 )* mutants at 22.5ºC were slightly decreased by the *ist-1* mutation (Figure 1a).

In contrast, and consistent with previous RNAi data (Wolkow, Munoz et al. 2002), we observed strong synthetic phenotypes when *ist-1 ( ok2706 )* was combined with mutations in *age-1* and *aap-1* genes. While the *age-1 ( hx546 ); ist-1 ( ok2706 )* double mutant exhibited no phenotype at 20ºC, it produced 50% sterile adults with undifferentiated germlines when raised at 25ºC (Figure 1b; Extended Data a). More strikingly, *ist-1 ( ok2706 )* double mutants with the stronger allele *age-1 ( mg305 )* showed a fully penetrant larval arrest phenotype at all tested temperatures (Figure 1c). The arrested larvae were dark-bodied L3 size, had few cells in the gonad primordium, lacked obvious radial and pharyngeal shrinkage and ceased pharyngeal pumping (Extended Data b). Moreover, *aap-1 ( m889 );* *ist-1 ( ok2706 )* double mutants showed no phenotype at 20ºC but developed as dauers at 25ºC, a phenotype not observed in either single mutant controls (Figure 1d). These findings were further corroborated using the *ist-1 ( gk5280 )* putative null allele (Cheng, Lee et al. 2022) (Extended Data c-e). Importantly, combining *ist-1 ( ok2706 )* with loss-of-function mutations in downstream components of the insulin pathway, *pdk-1 ( sa680 )* , *akt-1 ( mg306 )* and *akt-2 ( ok393 ),* did not result in major synthetic phenotypes (Figure 1e-g), except for a mild increase in dauer formation in *pdk-1 ; ist-1* mutants at 25ºC (Figure 1e) and a slight increase in sterile adults in *akt-1 ; ist-1* mutants at 27ºC (Figure 1f).

Collectively, these results support a role for IST-1 in larval development through the canonical *C. elegans* insulin pathway by promoting the activity of the phosphatidylinositol 3-kinase AGE-1 / AAP-1 , similar to what has been previously shown for IST-1 orthologues in mammals (Myers, Grammer et al. 1994) and *D. melanogaster* (Bohni, Riesgo-Escovar et al. 1999). To further support this conclusion, we combined *age-1 ( mg305 ); ist-1 ( ok2706 )* double mutants, which produce 100% arrested larvae at all temperatures (Figure 1c), with mutations in *daf-16* , *skn-1* , *hlh-30* and *hsf-1* genes that encode transcription factors known to transduce the signal from the insulin pathway (Lapierre, De Magalhaes Filho et al. 2013, Murphy and Hu 2013). As shown in Figure 1h, only loss of *daf-16* function was able to restore normal development in the *age-1 ( mg305 ); ist-1 ( ok2706 )* background.

Taken together, our data indicate that IST-1 signals through the canonical insulin pathway by modulating phosphatidylinositol 3-kinase activity (Figure 1i). It remains an open question whether IST-1 may also have a role in dauer formation via the parallel RAS pathway downstream DAF-2 (Nanji, Hopper et al. 2005) as previously suggested by Wolkow et al. using *ist-1* RNAi (Wolkow, Munoz et al. 2002). However, the complete suppression of *age-1 ( mg305 ); ist-1 ( ok2706 )* larval arrest phenotype by the *daf-16 ( mu86 )* mutation argues against a significant contribution of IST-1 to this parallel signaling branch.

## Methods

Embryo synchronization: To obtain a synchronized population of animals, approximately 20 gravid hermaphrodites were transferred to fresh NGM plates and allowed to lay embryos for 2-3 hours. Following this period, the parents were removed, leaving only the embryos on the plate, which were then incubated at the indicated temperatures.

Microscopy: *age-1 ( hx546 ); ist-1 ( ok2706 )* sterile adults and *age-1 ( mg305 ); ist-1 ( ok2706 )* arrested larvae were immobilized with 10mM levamisole and mounted on a slide with a 3% agarose pad. An Olympus BX61 fluorescence microscope equipped with a DP72 digital camera coupled to CellSens Software was used for image acquisition. Adobe Photoshop 2022 and Adobe Illustrator 2022 software were used to produce the figures.

Graphical and statistical analysis: Data were processed in Microsoft Excel and Prism GraphPad Software was used to generate the bar charts and perform statistical analysis.

## Reagents

**Table:** 

N2	*Wild type, DR subclone of CB original (Tc1 pattern I)*	CGC ^a^
BQ1	*akt-1 ( mg306 ) V¡ *	Patrick Hu gift
CB1370	*daf-2 ( e1370 ) III *	Gems et al. (1998) Genetics 150: 129-155
CF1038	*daf-16 ( mu86 ) I *	Lin et al. (1997) Science 278: 1319-1322
DR1567	*daf-2 ( m577 ) III *	Gems et al. (1998) Genetics 150: 129-155
DR2278	*aap-1 ( m889 ) I *	Manuel Muñoz gift
DR2290	*aap-1 ( m889 ) I; age-1 ( hx546 ) / mIn1 [ dpy-10 ( e128 ) mIs14 ( myo-2 ::GFP)] II *	Manuel Muñoz gift
JT9609	*pdk-1 ( sa680 ) X *	Paradis et al. (1999) Genes Dev. 13:1438-1452
PS3551	*hsf-1 ( sy441 ) I *	Hajdu-Cronin et al. (2004) Genetics 168: 1937-1949
QV225	*skn-1 ( zj15 ) IV *	Tang et al. (2015) G3 29: 551-558
RB2621	*ist-1 ( ok2706 ) X *	CGC ^a^
	*age-1 ( mg305 ) II *	Manuel Muñoz gift
TJ1052	*age-1 ( hx546 ) II *	Friedman and Johnson (1988) Genetics 118:75-86
VC204	*akt-2 ( ok393 ) X *	Patrick Hu gift
VC4195	*ist-1 ( gk5280 [loxP + P myo-2 ::GFP:: unc-54 3' UTR + Prps-27::neoR:: unc-54 3' UTR + loxP]) X *	CGC ^a^
VT1584	*hlh-30 ( tm1978 ) IV *	Grove et al. (2009) Cell 138: 314-327
VZ1001	*ist-1 ( ok2706 ) X *	This study, RB2621 6x outcrossed with N2
VZ1005	*daf-2 ( e1370 ) III; ist-1 ( ok2706 ) X *	This study, CB1370 x VZ1001
VZ1006	*daf-2 ( m577 ) III; ist-1 ( ok2706 ) X *	This study, DR1567 x VZ1001
VZ1009	*age-1 ( mg305 ) / mIn1 [ dpy-10 ( e128 ) mIs14 ( myo-2 ::GFP)] II *	This study, DR2290 x age-1 ( mg305 )
VZ1010	*akt-1 ( mg306 ) V; ist-1 ( ok2706 ) X *	This study, BQ1 x VZ1001
VZ1043	*age-1 ( hx546 ) II; ist-1 ( ok2706 ) X *	This study, TJ1052 x VZ1001
VZ1055	*age-1 ( mg305 ) / mIn1 [ dpy-10 ( e128 ) mIs14 ( myo-2 ::GFP)] II; ist-1 ( ok2706 ) X *	This study, VZ1001 x VZ1009
VZ1103	*aap-1 ( m889 ) I; ist-1 ( ok2706 ) X *	This study, DR2278 x VZ1001
VZ1118	*age-1 ( mg305 ) / mIn1 [ dpy-10 ( e128 ) mIs14 ( myo-2 ::GFP)] II; hlh-30 ( tm1978 ) IV; ist-1 ( ok2706 ) X *	This study, VT1584 x VZ1055
VZ1123	*pdk-1 ( sa680 ) ist-1 ( gk5280 [loxP + P myo-2 ::GFP:: unc-54 3' UTR + Prps-27::neoR:: unc-54 3' UTR + loxP]) X *	This study, JT9609 x VC4195
VZ1145	*hsf-1 ( sy441 ) I; age-1 ( mg305 ) / mIn1 [ dpy-10 ( e128 ) mIs14 ( myo-2 ::GFP)] II; ist-1 ( ok2706 ) X *	This study, PS3551 x VZ1055
VZ1150	*daf-16 ( mu86 ) I; age-1 ( mg305 ) / mIn1 [ dpy-10 ( e128 ) mIs14 ( myo-2 ::GFP)] II; ist-1 ( ok2706 ) X *	This study, CF1038 x VZ1055
VZ1251	*age-1 ( mg305 ) / mIn1 [ dpy-10 ( e128 ) mIs14 ( myo-2 ::GFP)] II; skn-1 ( zj15 ) IV; ist-1 ( ok2706 ) X *	This study, QV225 x VZ1055
VZ1154	*akt-2 ( ok393 ) ist-1 ( gk5280 [loxP + P myo-2 ::GFP:: unc-54 3' UTR + Prps-27::neoR:: unc-54 3' UTR + loxP]) X *	This study, VC204 x VC4195
VZ1328	*age-1 ( hx546 ) II; ist-1 ( gk5280 [loxP + P myo-2 ::GFP:: unc-54 3' UTR + Prps-27::neoR:: unc-54 3' UTR + loxP]) X *	This study, TJ1052 x VC4195
VZ1331	*aap-1 ( m889 ) I; ist-1 ( gk5280 [loxP + P myo-2 ::GFP:: unc-54 3' UTR + Prps-27::neoR:: unc-54 3' UTR + loxP])/+ X *	This study, DR2278 x VC4195
VZ1338	*age-1 ( mg305 ) II; ist-1 ( gk5280 [loxP + P myo-2 ::GFP:: unc-54 3' UTR + Prps-27::neoR:: unc-54 3' UTR + loxP]) X *	This study, VZ1009 x VC4195

^a^ CGC: Caenorhaditis Genetics Center

## Data Availability

Description: Images of sterile and arrested animals and graphs with ist-1(gk5280) allele. Resource Type: Image. DOI: https://doi.org/10.22002/rs6tg-ejg41
